# Face-to-face contact during infancy: How the development of gaze to faces feeds into infants’ vocabulary outcomes

**DOI:** 10.3389/fpsyg.2022.997186

**Published:** 2022-10-28

**Authors:** Zsofia Belteki, Carlijn van den Boomen, Caroline Junge

**Affiliations:** Department of Experimental Psychology, Helmholtz Institute, Utrecht University, Utrecht, Netherlands

**Keywords:** faces, social development, infancy, gaze, vocabulary

## Abstract

Infants acquire their first words through interactions with social partners. In the first year of life, infants receive a high frequency of visual and auditory input from faces, making faces a potential strong social cue in facilitating word-to-world mappings. In this position paper, we review how and when infant gaze to faces is likely to support their subsequent vocabulary outcomes. We assess the relevance of infant gaze to faces selectively, in three domains: infant gaze to different features within a face (that is, eyes and mouth); then to faces (compared to objects); and finally to more socially relevant types of faces. We argue that infant gaze to faces could scaffold vocabulary construction, but its relevance may be impacted by the developmental level of the infant and the type of task with which they are presented. Gaze to faces proves relevant to vocabulary, as gazes to eyes could inform about the communicative nature of the situation or about the labeled object, while gazes to the mouth could improve word processing, all of which are key cues to highlighting word-to-world pairings. We also discover gaps in the literature regarding how infants’ gazes to faces (versus objects) or to different types of faces relate to vocabulary outcomes. An important direction for future research will be to fill these gaps to better understand the social factors that influence infant vocabulary outcomes.

## Introduction

Early word learning appears a difficult task, given that an infant’s environment is full of objects and sounds (Quine, 1960; Smith and Yu, 2008). Infants acquire words from the people they interact with, who repeatedly expose them to certain word-to-world combinations. The first signs of infants’ word comprehension are observable as early as 6 months (Bergelson and Swingley, 2012; Tincoff and Jusczyk, 2012). This precedes the earliest observations of more complex social abilities that are first observed 9 months of age onward, such as joint attention, and which are considered instrumental for word learning (Carpenter et al., 1998; Cleveland et al., 2007). Previous literature has frequently shown that where infants attend in such social settings can have cascading effects on how their word learning progresses (Moore et al., 1999; Charman et al., 2000; Mundy et al., 2003; Slaughter and McConnell, 2003; Houston-Price et al., 2006). One such powerful cue could be how infants attend to other faces present in the interaction (from now on “gaze to faces”; Hessels, 2019), as infants start attending to faces and to features within faces very early on in life (Grossmann, 2017). The purpose of this position paper is to review studies demonstrating a link between infant gaze to faces and vocabulary outcomes. We will assess the literature on gaze to faces, because it is considered one of the pre-requisites of later developing more complex social abilities [such as following the gaze of a social partner (Reid and Striano, 2005; Gredebäck et al., 2010; Grossmann, 2017)] that are found to correlate with word learning and vocabulary outcomes. Our aims in this position paper are to find new directions for future research to improve our understanding on whether, when, and how gaze to faces feeds into vocabulary acquisition.

While the relevance of being able to attend to social partners has been acknowledged for all facets of early language development, it is likely that gaze to the faces and facial features of social partners helps to bootstrap one aspect of language acquisition in particular: vocabulary acquisition (Tomasello, 1992, 2000; Çetinçelik et al., 2021). Acquiring a vocabulary requires the mapping of auditory spoken words to their matching concepts, both of which may require some level of disambiguation. The words that infants tend to acquire early are concrete objects that are visually present in the interaction, such as “bottle” and “sock” (Kavanaugh and Jirkovsky, 1982; Bergelson and Swingley, 2012; Braginsky et al., 2019). Infants can benefit from attending to the face of their social partner, as it can provide both visual (e.g., their eyes gazing at the named object) and auditory cues (e.g., mouth speaking the word form) to guide word learning. We theorize that infant gaze to faces is important to vocabulary acquisition, not only as it is a precursor to infants’ ability to utilize the cues that faces signal to word learning, but also as it scaffolds the development of those more complex social abilities instrumental in word learning (e.g., gaze following, joint attention, Reid and Striano, 2005). These more complex social abilities have been shown to relate to vocabulary outcomes; however, the relation between the precursor of these complex social abilities and vocabulary outcomes has been contemplated less (Carpenter et al., 1998; Morales et al., 2000; Slaughter and McConnell, 2003). Therefore, we examine the relevance of infant gaze to faces by zooming in on vocabulary as our chosen outcome measure. Note that vocabulary outcomes can refer to both productive and receptive vocabulary. Whenever there are mixed findings in the literature discussed below, we highlight the nature of the vocabulary outcomes (i.e., whether expressive, receptive, or both vocabulary outcomes are affected), as this distinction may help to explain mixed results.

The relevance of gaze to faces to vocabulary is not yet fully understood. One complexity stems from the gaze to faces being defined in a number of ways. For instance, we can assess it by zooming in on the relevance of specific elements of a face, or we can compare it against other objects present within the same scene, or by contrasting different kinds of faces across different interactions. Moreover, it remains unclear whether this relationship proves stable across development or whether there are any developmental trends. It could be that the relevance of gaze to faces wanes off across development, but holds especially for the youngest age group just starting to build a vocabulary but who cannot yet rely on higher social–cognitive abilities such as joint attention. It is also unclear whether the choice of experimental tasks impacts the relationship. That is, it is possible that how infants utilize and weigh cues in a face may depend on the situation at hand and their developmental stage, making it difficult to understand exactly the relationship between infant gaze to faces and vocabulary outcomes. For instance, in cases where it may be relatively difficult to hear the words correctly, infants may look more at the mouth, whereas in cases where there are multiple novel objects present, infants may benefit more from gazing at the eyes. Hence, the relevance that looking to the mouth will have to infants’ word learning and subsequent vocabulary outcomes will depend on what needs to be disambiguated in the task, and whether the infant is at a developmental level where they can successfully disambiguate this information. Considering this, the developmental stage of the infant and the task they are presented with are important to consider in our review, as they may moderate which aspects of gaze to face are the most related to vocabulary outcomes.

This paper is organized as follows. We will start by presenting an overview of the existing theoretical frameworks pointing to gaze to faces as a possible facilitator to vocabulary outcomes in infancy. Then, we will review relevant literature that assesses how gaze to faces impact vocabulary outcomes, for three specific aspects of gaze to faces: first, to specific elements in the face (the eyes compared to the mouth); then, to faces versus other elements in the environment; finally, to different types of faces. Regarding vocabulary outcomes, we will assess papers look at both productive vocabulary outcomes and receptive vocabulary outcomes. Within each section, We will assess the relevance of this specific type of gaze to faces to vocabulary outcomes and describe how this relation is moderated by developmental trends and experimental tasks. In our discussion, we will evaluate the evidence for and against a relation between gaze to faces and vocabulary outcomes, based on the literature reviewed. We will then conclude by addressing the questions that are left unanswered by the existing literature, followed by recommendations for future research directions.

We therefore aim not only to collate literature showing whether, when, and why infant gaze to other persons’ faces could scaffold infants’ vocabulary outcomes (Locke, 1993) but also to identify gaps in the literature. Our overarching goal will be to complement and bridge existing theoretical frameworks that link social factors to early word learning (Tomasello, 2000; Werker and Curtin, 2005; Kuhl, 2007; Çetinçelik et al., 2021; Bastianello et al., 2022).

## Theoretical frameworks on infancy: Why would gaze to faces facilitate vocabulary?

Research has evidenced the crucial role that the social environment has in language acquisition in the first years of life (for a review, see e.g., Kuhl, 2007). Similarly, theoretical frameworks have attempted to outline the feedforward effects of the social environment on language acquisition (Pinker, 1979; Hollich et al., 2000; Tomasello, 2000; Werker and Curtin, 2005; Chater and Manning, 2006; Kuhl, 2007; Frank et al., 2009a; Gogate and Hollich, 2010; Johnson, 2016). This section aims to outline the theoretical frameworks that link the dynamics of the social environment to vocabulary acquisition to provide the motivation for assessing the feedforward effect that infant gaze to faces may have on their vocabulary outcomes.

The social environment is composed of many sub-parts of parental warmth and input (Madigan et al., 2019; Anderson et al., 2021). To facilitate word learning, it is beneficial to infants to detect the communicative intent from the partner to the infant, as it highlights potential situations where infants can acquire new words (Floor and Akhtar, 2006). One important source for detecting communicative intent is provided by the faces of social partners. Cues, such as direct gaze (but not averted gaze), can indicate to the infant that their social partner wishes to engage with them (Senju and Csibra, 2008). Indeed, one account on infant language development, the Social-Pragmatic account of language acquisition, holds that infants’ ability to recognize communicative intent is crucial to language learning and more specifically their learning of words (Tomasello, 1992, 2000). This theory considers the detection of communicative intent critical, as it provides the foundation of more complex social behaviors (such as joint attention) that are required for language learning. Hence, since the Social-Pragmatic account holds that recognizing communicative intent mediates word learning, it follows that infants’ gazes to faces as the more socially informative parts of the environment could be related to their vocabulary outcomes.

Besides detecting the cues that signal communicative intent, infants need to be able to combine and comprehend information that is presented multi-modally in order to map meaning to words. That is, infants need to map the auditory (i.e., words) information to the visual (i.e., concrete objects present in the scene) information. As is highlighted in the Intersensory Redundancy hypothesis, learning is facilitated whenever there is temporal synchrony between two modalities, because the same amodal information is highlighted above other sources of non-synchronous amodal information (Bahrick and Lickliter, 2012). While this account is not specific to word learning, it predicts that multi-modal information, as is present in dynamic faces, would facilitate word learning by linking information from two modalities through their temporal synchrony. That is, faces provide information from two modalities at the same time: The auditory information that we hear and the visual information from the movements of the eyes and mouth. Therefore, an early face preference is arguably necessary to selectively direct the attention of the infant. This then allows them to gain extensive experience with faces and thereby successfully learn to process multimodal information presented by their environment. For example, viewing a moving mouth and hearing speech involves information from the audio and visual modalities that can be linked based on their temporal synchrony to create a combined auditory and visual signal, i.e., a speaking mouth producing sounds, that benefits word recognition (Hollich et al., 2005). Infants may use this synchronous information not only to improve word processing (the mouth-word relationship) but also to facilitate the word-to-world mappings (using visual cues such as the speaker’s gaze to a named object, the eyes-word relationship) (Gogate and Hollich, 2010; Bahrick and Lickliter, 2014). Therefore, how well infants can connect the auditory to visually synchronous information that they receive from faces may relate to their vocabulary outcomes.

Above, we discussed two theories that emphasized that gaze to faces could provide useful cues to guide infants’ word learning. The theories posited that gaze to faces could improve awareness of communicative intent, boost auditory word processing, and guide word-object pairings, all of which are instrumental for the learning of words. In both the Social Pragmatic Account and the Intersensory Redundancy hypothesis, it was argued that infants need to be able to flexibly utilize the multiple cues that they receive from social partners to learn words (Tomasello, 2000; Bahrick and Lickliter, 2014). But will there be development when infants start utilizing these cues? There are two theoretical frameworks on infant language learning that emphasize that there is a progression in the kind of cues that infants use to guide their early word learning: the PRIMIR framework (a developmental framework for Processing Rich Information from Multi-dimensional Interactive Representations; Werker and Curtin, 2005) and the ECM-model (Emergentist Coalition Model; Hollich et al., 2000). Both accounts acknowledge that the extent to which infants will be able to utilize cues will depend on the developmental stage of the infant. PRIMIR focuses on explaining development in speech perception and word learning by progression in the developmental level of the child, next to initial biases and language-specific requirements. The ECM-model stresses more the social nature of word learning, as it explains development in word learning through the combined roles of social-pragmatic factors, cognitive constraints, and global attentional mechanisms (Hollich et al., 2000; see also Tomasello, 2000 for the social-pragmatic account). As we are interested in gaze to faces as a social cue likely to facilitate vocabulary acquisition, we examine this account more closely below.

The Emergentist Coalition model creates an important distinction between cues that are available versus utilized by infants, arguing that younger children rely on only a subset of the cues that older children are able to use. For example, whereas 7–8-month-old infants are shown to have their word recognition disrupted if a familiarized word is produced by a new voice, at 11 months infants no longer show this disruption (Houston and Jusczyk, 2003). Thus, although the social environment makes a number of cues available (such as multiple speakers repeating the same word; McRoberts et al., 2009), infants’ utilization of these cues is dependent on their cognitive and social abilities. That is, attending to socially informative information present in faces will relate to word learning if (a) the infant is at a developmental stage where they are able to use the socially informative information for word learning, and (b) the information is relevant to the task at hand, and thus indeed informative. Therefore, whether gaze to faces proves relevant to vocabulary outcomes may depend on experimental parameters, such as ages tested or choice of tasks. For instance, since we know that certain social abilities, such as joint attention and gaze following (Reid and Striano, 2005), become relevant to vocabulary acquisition later in development, we can similarly imagine that the relevance of gaze to face to vocabulary acquisition also changes over development. Across development and across tasks, we may expect different relations between infant gaze to faces and vocabulary outcomes. This is why we next turn to review empirical evidence not only on whether gaze to faces feeds into vocabulary, but also to evaluate how this relation is affected by the developmental stage of the infants and tasks at hand.

## How does gaze to faces relate to vocabulary outcomes?

The faces of social partners can provide infants with cues to guide their leaning of words but can be indexed in different ways. We will relate three specific aspects of gaze to faces relevant to vocabulary outcomes: first, gaze to specific facial features – to the eyes versus the mouth: then selective gaze to faces relative to other objects; and finally, selective gaze to more versus less social faces. In each of these three subsections, we will relate gaze to faces to vocabulary outcomes across infants’ developmental trajectories and tasks.

### How do gaze to the eyes and the mouth relate to vocabulary?

During our literature review, we found multiple studies that related infants’ fixations to the eyes and mouth to vocabulary outcomes (for recent reviews, see Çetinçelik et al., 2021; Bastianello et al., 2022). In what follows next, we first zoom in on whether there is any development across infancy in how infants attend to those facial features. For each facial element, we then consider how this relation to vocabulary is moderated by development and task. Finally, we explain the observed patterns by returning to the theories discussed earlier.

When infants are exposed to faces, they usually fixate first on the eyes of a social partner (Hills et al., 2013). Additionally, they attend to the eyes longer and more frequently than to the mouth (Haith et al., 1977). Infants prefer to look at eyes, even when presented with faces missing various parts (e.g., eyes, mouth, or nose): 2-month-olds fixate equally long to complete faces as to faces with only the eyes are present, and less to faces with only the mouth or the nose present (Maurer, 1985). From 2 to 6 months of age infants fixate more to the eyes than on other features of a social partner including their mouth and body (Jones and Klin, 2013). Some studies indicate that infants maintain the highest proportion of gaze to the eyes (Hunnius and Geuze, 2004; Lewkowicz and Hansen-Tift, 2012; Morin-Lessard et al., 2019). Others find that this higher frequency of fixations to the eyes is only to be present earlier in infancy (Frank et al., 2012). Although some studies indicate infants have a robust preference to attend to eyes, the mixed findings of studies indicate that this does not necessarily hold across development or across tasks.

Although the eyes remain the primary focus of attention, the mouth also increasingly draws attention during the first year of life (Young et al., 2009; Lewkowicz and Hansen-Tift, 2012; Tenenbaum et al., 2013; Elsabbagh et al., 2014). This finding is confirmed by several studies, notably with high participant numbers, and thus with good statistical power. These studies often test infants at multiple time points throughout the first years of life, some looking at as many as four to five separate developmental time points (Lewkowicz and Hansen-Tift, 2012). Infants are observed, in a task where they are gazing at a speaking face, to look longer to the eyes at 4 months, equally to the eyes and mouth at 6 and 8 months, more to the mouth at 10 months and finally more to the eyes at 12 months (Lewkowicz and Hansen-Tift, 2012). Therefore, across different timepoints in the first year of life, infants divide their length of fixations between the eyes and the mouth differentially for the same task.

The developmental stage of the infant also impacts the frequency with which infants attend to different types of eyes and mouths. That is, infants show progression in the types of facial features they prefer to attend to. For example, although both 9- and 10-month-old infants can differentiate between open and closed eyes, it is only from 10 months onward that infants recognize that only open eyes could provide information about where a social partner is looking (Brooks and Meltzoff, 2005). Thus, infant sensitivity to open versus closed eyes is a prerequisite to being able to follow their partner’s eye gaze, which is a powerful cue shown to predict subsequent productive vocabulary growth (e.g., (Brooks and Meltzoff, 2008, 2015). This indicates a developmental progression in the extent to which infants can utilize the cues provided by the eyes to direct their gaze.

There are also noticeable changes in how infants attend to different types of mouths. Infants increasingly also prefer looking at speaking mouths over other types (such as smiling mouths) from 6 to 9 months of age (Tenenbaum et al., 2013, 2015). Another example is that infant gaze to facial elements hinges on the type of speech they hear: a cross-sectional study manipulating whether infants watched speakers’ producing a language that was native versus non-native to the infant showed that the looks that infants directed to the eyes and the mouth differed across the native and non-native speech conditions. While 4- to 8-month-olds increasingly devoted more gazes to the speakers’ mouths irrespective of the type of speech, 12-month-olds only fixated more to the mouth when they heard a speaker producing a non-native versus native language (Lewkowicz and Hansen-Tift, 2012). This finding is observed cross-linguistically, with infants also undergoing an attentional shift to the mouth when the dominant language in their environment is Japanese (Sekiyama et al., 2021).

Another study manipulated whether or not there was synchrony between audio and visual information: at 10 months, infants’ usual pattern of a preference to fixate to a mouth was absent in the desynchronized condition compared to when synchronized audio-visual information was presented (Hillairet de Boisferon et al., 2017). This finding held both when infants were presented with native or non-native speech. Similarly, looking times to the mouth were longer when infants were presented with speaking compared to silent faces – in the latter condition, looking times to the eyes were shown to be significantly longer (Tomalski et al., 2013). All these illustrations thus point to infants becoming increasingly sensitive to those situations that prove maximally informative, and this is mirrored in their differential gazes to specific facial regions.

While above we summarized studies that provide evidence of a development in how infants attend to both the eyes and the mouth, two recent review papers provide ample evidence that gaze to eyes (Çetinçelik et al., 2021) as well as a gaze to the mouth prove relevant to vocabulary outcomes (Bastianello et al., 2022). We complement their reviews by focusing on those studies that assess whether there is the developmental change in the relevance of gaze to eyes versus mouth regions.

The review by Bastianello et al. (2022) confirmed that increased gaze to (speaking) mouths around the first year of life is associated with infants’ early expressive language skills across all of the reviewed studies in the paper. Yet, for slightly younger infants (5-month-olds), it is infant gaze to the eyes (over the mouth) that has been shown to predict their receptive vocabulary size at 14 months (Viktorsson et al., 2021). Similarly, in a situation where the face provides mismatched auditory and visual information, it is again longer gaze times to the eyes and shorter gaze times to the mouth that correlates positively with 6 to 9-month-old infants’ later receptive and productive vocabulary outcomes (Kushnerenko et al., 2013). However, in another study presenting infants with mismatched auditory and visual information, infants are found to have increased looking times to the mouth between 6 and 9 months of age (Tomalski et al., 2013). This seemingly contradictory pattern of results can be explained by the observation that while infants increasingly attend to the mouth over the course of development, they may not directly be able to utilize all cues that the speaker’s mouth provides (Hollich et al., 2000). Given that integration of multi-sensory information is underdeveloped at birth and develops over the course of the first year (Burnham and Dodd, 2004; Bahrick et al., 2013), it could be that these younger infants could not yet fully utilize the multi-modal information most saliently present in the mouth region (Nardini et al., 2010). In this case, younger infants’ capacity to ignore mismatched an unreliable cues and to rely more on the cues of the eyes instead may be predictive of vocabulary outcomes (Kushnerenko et al., 2013). In comparison, older infants may be able to use multimodal (that is, auditory and visual) information more flexibly, including in situations where the modal information is mismatched. Thus, there is development in how informative facial features can be to infants and subsequently in how infants’ gazes to the eyes and mouth relate to vocabulary outcomes.

While most research points to a positive link between infants’ increased fixation to the mouth or eyes with vocabulary outcomes, it is important to consider that this relation may not only hold for specific ages, but also for specific tasks (Kushnerenko et al., 2013; Altvater-Mackensen and Grossmann, 2015; Ter Schure et al., 2016; Danielson et al., 2017; Bastianello et al., 2022). To illustrate the effect of the task, we compare studies that differed in the complexity of the presented scene, while also linking infant gaze to vocabulary outcomes. In visually complex scenes (live action scenes with many characters performing different activities), 7-month-olds’ increased fixations to the mouth is shown to be associated with superior productive vocabulary outcomes at 36 months (Frank et al., 2012). In comparison, in simpler live-action scenes (that contain a single face displaying communicative signals), increased fixations to the mouth in contrast relate to inferior productive vocabulary (Elsabbagh et al., 2014). Why might this be? This could be because the two tasks indicate different abilities when the infants fixate to the mouth. In the simpler scenes, a speaking mouth is the most perceptually salient feature within the scene, whereas in the visually complex scenes, a speaking mouth competes with other perceptually salient elements, but is still the most cue relevant for vocabulary. It is therefore important to consider how the same behavior (e.g., gaze to the mouth) may indicate different abilities depending on the task with which the infant is presented, and which cue (within the task) happens to be the most relevant for word learning. When evaluating how infants’ gazes to faces relate to vocabulary outcomes, it is therefore important to evaluate which cue is likely to be the most informative to the infants’ word learning.

Having above reviewed evidence that both gaze to eyes as well as mouth prove relevant to vocabulary outcomes, we now turn to explain why this could be. Infants’ fixations to the eyes could be beneficial to their word learning because the eyes of a social partner can signal their communicative intent or provide information about the referent to which a social partner is attending (Tomasello, 1992, 2000). Some studies show that infants first rely more on attentional cues, such as perceptual salience, rather than on gaze-cues from the interlocutor to guide their early word-object mappings (Brooks and Meltzoff, 2005, 2015; Pruden et al., 2006). As eyes are a perceptually salient feature of the face to which infants have a bias to attend to, then eyes may be one of the features that in early infancy draws infants to attend more to faces than to other objects (Di Giorgio et al., 2012). As an example, one study shows that infants who are able to detect when they are being gazed at and who can also subsequently follow the partner’s gaze direction to an object may have a considerable advantage in determining the thoughts and intentions of their social partners (Langton et al., 2000). This is advantageous for word learning because understanding the internal state of a social partner increases the probability of an infant correctly discerning which of the many possible referents a social partner is communicating about.

While the eyes provide visual information to whom or about what the speaker is communicating, the mouth provides multimodal (visual and auditory) information about what is being said. In line with the Intersensory Redundancy Hypothesis, the combination of auditory and visual information may benefit word learning in multiple ways (Bahrick and Lickliter, 2012; Lewkowicz and Hansen-Tift, 2012). The development of increased fixations to mouths may benefit word learning because the mouth can provide a combination of auditory and visual cues that aid in the learning of words. This audio-visual information allows listeners to narrow down potential words by segmenting speech streams and locating word boundaries in continuous speech (Hollich et al., 2005; Mitchel and Weiss, 2014). Thus, the synchrony of the visual and auditory modalities (i.e., the movements of the mouth combined with the sounds it produces) makes it easier to narrow down *what* is being said (thereby facilitating infants’ receptive vocabulary), *how* it is said (thereby facilitating infants’ learning of expressive vocabulary), and *who* said it (highlighting communicative intent) (Bahrick and Lickliter, 2012; Lewkowicz and Hansen-Tift, 2012; Benders, 2013; Altvater-Mackensen and Grossmann, 2015). Infants who are learning words may therefore benefit from fixating to the mouth as a way to reduce several kinds of ambiguities of the visual and/or auditory information they are receiving through the combination of the two modalities.

Besides which facial elements infants selectively attend to, this review further demonstrates that the developmental stage of the infant plays a large role. We synthesized research reporting that progression in how infants attend to eyes versus mouth was relevant to their vocabulary acquisition. According to the Emergentist Coalition model, how infants develop their detection of and subsequently utilize socially more informative cues (such as speaking mouths) is critical to word learning (Hollich et al., 2000). In line with this, infants’ development in the utilization of the social cues provided by the eyes is shown to correlate with later receptive and productive vocabulary (Brooks and Meltzoff, 2005, 2008). Additionally, the shift in gaze to specific types of mouths, i.e., speaking ones, is also shown to relate to vocabulary outcomes; for example, 6-month-olds who fixate more to the mother’s mouth during live interaction have the superior productive vocabulary at 24 months (Young et al., 2009). This preferential fixation to speaking versus silent mouths could be beneficial to word learning by increasing the likelihood that the infants fixate to a mouth from whom they can learn words. Fixating more frequently to certain eyes and mouths may in turn facilitate word learning by increasing the likelihood of infants’ fixating to a partner who is providing more communicative cues (e.g., one with direct gaze and/or a speaking mouth) and thereby increasing the opportunities for word learning.

### How does gaze to faces relative to other objects relate to vocabulary?

In the examination of the literature that preceded the writing of this review, we could not find empirical evidence that assessed the impact that infants’ preferential gaze to faces relative to objects had on their vocabulary outcomes. However, we theorize that infants’ gazes to faces relative to other objects is a pre-requisite to more complex social abilities (such as gaze following), which have been shown to be linked to vocabulary outcomes (Carpenter et al., 1998; Morales et al., 2000; Slaughter and McConnell, 2003). We theorize this because it appears logical that before infants can follow adults’ gaze correctly to labeled objects, infants first require ample experience with faces. This experience may be facilitated by the presence of a preference for faces over other objects. In this section we therefore review only the evidence of whether there are developmental changes in infant gaze to faces, and whether there are task-related changes. We then continue to speculate how this gaze to faces relative to other objects may relate to infants’ vocabulary outcomes and how this relation may change across development and tasks.

Throughout infancy, infants are shown to have a preferential bias to attend to faces compared to other objects, looking longer to and orienting more frequently to face-like stimuli compared to non-face-like stimuli (Johnson et al., 1991; Valenza et al., 1996). This is observed across a number of visual attention tasks: some studies reporting a face-preference involve infants’ free viewing of clips that contain faces (e.g., video clips from the TV show Sesame Street) (Frank et al., 2009a,^2014^; Franchak et al., 2016). Other studies look at preferential biases for faces by presenting images of faces together with other static objects (e.g., birds and cars) (Gliga et al., 2009; Elsabbagh et al., 2013). In the case of both paradigms, studies calculate the percentage of trials where the first fixation is directed to a certain category of object and/or calculate the average number of fixations to an area of interest. These similarities in how studies define infants’ gaze to faces make it easier to cross-compare findings across studies (Hessels, 2019). In both paradigms, studies find that infants tend to direct their first gazes to faces as well as fixate more frequently to faces, compared to other stimuli on the screen (Gliga et al., 2009; Elsabbagh et al., 2013). Based on the qualities (e.g., sufficient participant numbers) and reoccurring findings of the studies, the finding that infants prefer to attend to faces (compared to other objects) appears robust.

Although all tasks show that infants primarily attend to faces, studies differ in the proportions of face preference, possibly based on differences in study parameters, such as children’s age and children’s opportunities to explore. To illustrate the mixed findings for the effect of age, there is a set of studies that recorded fixations to all available visual stimuli in a natural interaction for infants between 1 and 24 months of age. These studies report that across age, there is a decline in the frequency of gaze to faces, coupled with an increasing frequency of gaze to hands, meaning that age has an effect on the frequency of gazes to faces versus hands (Jayaraman et al., 2015; Fausey et al., 2016). In this paradigm, infants freely move about in their home environment while their gaze patterns are measured with head-mounted eye-trackers. Contrastingly, other studies (using different tasks) suggest that older infants fixate to faces more than younger infants do when watching video clips (6–24-month-olds; Franchak et al., 2016; 3–9-month-olds: Frank et al., 2014). The mixed findings across different tasks make it important to consider which aspects of the task or situation led to differences in infants’ observed frequency of fixations to faces. Factors may include aspects of the methodology such as contrasting stimuli presented or whether the infants’ movements during eye-tracking are restricted (as in Frank et al., 2014; Franchak et al., 2016) or not (as in Jayaraman et al., 2015; Fausey et al., 2016). Overall, it appears that the frequency of fixations that infants direct to faces hinges on their capacity for movement. Infants’ capacity for movement depends both on their developmental stage (i.e., whether they can walk or sit up) and the task at hand (i.e., whether the procedure restricts their movements or not). Regarding developmental stage, older infants (who are more mobile) are shown to receive a more mixed visual input on faces and hands, whereas younger infants receive more input from faces. Regarding tasks, infants’ preference for faces may be stronger, when their movement and visual input is restricted. When drawing conclusions on the development and ubiquity of face preferences, it is therefore important to consider how the methodological choices may impact the behaviors that infants display during the procedure.

In the preceding paragraphs, we have considered how the developmental stage and task at hand influence how infants fixate to faces. Will this type of gaze to faces (face preference) also impact their vocabulary? As we have seen in the theoretical accounts, fixating to faces can be a facilitator of word learning because the cues provided by the face allow infants discern what their partner intends to communicate about and to whom their communication is directed to, e.g., to the infant, thereby guiding the infants’ learning of words. Indeed, the bias to fixate to faces (over other objects) is arguably an important prerequisite to infants’ vocabularies because it directs infant gaze to the cues provided by the face (Tomasello, 1992, 2000). Yet it is likely that there are developmental patterns as we have seen that face preference changes with development. A higher proportion of fixations to faces compared to other objects could be particularly important in younger infants, who have less developed social and cognitive abilities than older infants (Hollich et al., 2000), which in turn may compromise their ability to direct and maintain their gaze to objects in their environment, and as a result make them more dependent on a social partner to guide their learning (Colombo and Cheatham, 2006; Reynolds et al., 2013). Future research could investigate whether the relevance of preferential fixation to faces to vocabulary outcomes is more substantial early in vocabulary development, but declines with age. Furthermore, future research should consider how the choice of paradigm affects the relation between preferential fixation to faces and vocabulary outcomes.

### How does gaze to more social versus less social faces relate to vocabulary?

When we are considering the types of faces that might prove informative, we note that current studies have not correlated infants developing a preference for more social faces to their vocabulary outcomes. Just as in the preceding subsection, we therefore first evaluate whether there are potential meaningful changes in this type of gaze to faces before, we speculate whether this could impact infant vocabulary outcomes.

Infants gradually begin to preferentially attend to certain types of faces over others, that is, faces that contain potentially more social cues (Smith and Gasser, 2005; Frank et al., 2009b,^2014^; Slater et al., 2010). From 3 months onward, infants begin to prefer natural face images to unnatural ones (Turati et al., 2005). Around this age, they are also shown to preferentially fixate to upright over inverted faces (Chien, 2011; Elsabbagh et al., 2013). By 6 months, infants direct their first gazes to upright and not to inverted faces (Gliga et al., 2009). Additionally, infants are shown to develop a preference to fixate to speaking faces above silent dynamic faces from 2 to 8 months, such that older infants increase their looks to speaking faces and decrease their looking away rates (Bahrick et al., 2016). Thus, the development of preference for specific faces appears to be robust across tasks; infants’ increased sensitivity to certain faces moves toward faces to which infants are frequently exposed in their daily environment. Infants’ gazes also increase toward more communicative faces over the course of development, while their gazes to less communicative faces remain similar (Bahrick et al., 2016). This preference to specific types of faces could be an index of perceptual learning, which refers to an increased sensitivity to specific faces frequently present in the environment and decreased sensitivity to others (Maurer and Werker, 2014).

When we are considering the relevance of attending to faces that are more social than other faces, we can only speculate that preferential gaze to more social faces could relate to vocabulary outcomes. Social faces are those types of faces that provide more explicit cues indicating communicative intent directed at the child (e.g., *via* direct gaze), and which are present often in the child’s environment. As is theorized in the Social Pragmatic account of word learning, the understanding of communicative intent is key to the learning of words (Tomasello, 1992, 2000). Whether infants gaze to more social faces (that contain more explicit cues) arguably increases their opportunities for word learning and subsequently relates to their vocabulary outcomes. The relation between gaze to social faces and vocabulary outcomes may hold in particular for younger infants. Whereas older infants are shown to be able to learn words by overhearing conversations, it is less clear whether younger infants have similar capacities (Akhtar et al., 2001). Younger infants may rely more on communicative intent recognized by more visual engagement with the speaker (Tomasello, 2000). Therefore, infants’ capacities to attend to more socially relevant faces could relate more to vocabulary outcomes when they are younger.

There are other task-related factors which arguably influence how socially relevant certain types of faces are, and which further could impact vocabulary outcomes. For example, whether or not a person’s face is physically present or whether this person is reciprocating the infants’ behaviors could ultimately influence vocabulary outcomes. Whereas some studies find that infants can learn from digitally presented faces (i.e., faces that are not physically present) that direct their gaze at a target object (Houston-Price et al., 2006), others find that there is a “video-deficit,” and that infants’ learning is hindered when the tutor appears on video instead of being physically present (Anderson and Pempek, 2005). Findings are mixed regarding the extent to which infants can use the cues provided by physically versus digitally presented faces to guide their word learning (O’Doherty et al., 2011; Roseberry et al., 2014; Troseth et al., 2018; Tsuji et al., 2020). In part, these mixed findings may also depend on the difficulty of the word-learning task with which the infant is presented (Gogate and Madhavilatha, 2017). More difficult word-learning tasks may benefit from the faces of social partners being physically present and reciprocating. Further research is needed to clarify in which tasks infants can use the face to guide their learning of words. Additionally, further research is required as to how the difficulty of the word-learning task impacts infants’ abilities to learn from faces that are not physically present and/or not reciprocating.

To summarize, based on the existing literature, it remains unclear how infants’ fixation toward more socially relevant faces impacts their vocabulary outcomes. Further research is needed to elucidate how flexibly infants can learn word-world pairings from the faces of social partners and the extent to which this flexibility depends on the developmental stage of the infant, and the task at hand.

In the above three sections of this paper, we discussed how three aspects of infant gaze to faces relate to vocabulary outcomes: gaze to different elements within faces; gaze to faces relative to other objects; and gaze to different types of faces. We will now discuss our findings in the literature and make suggestions for future research.

## Discussion

This position paper first aimed to assess how infant gaze to faces may feed into their vocabulary outcomes. We reviewed the literature on three aspects of infant gaze to faces: gaze to the eyes compared to the mouth; gaze to faces compared to objects; and gaze to more socially relevant faces. Several studies were found that related infant gaze to facial elements and vocabulary outcomes. Here, we observed that the relationship between infants’ gaze to eyes and the mouth with their vocabulary outcomes was impacted by the developmental stage of the infant, and the task at hand. However, to the best of our knowledge, no studies explored how gaze to faces (compared to other objects) or gaze to more social (compared to less social) faces relate to vocabulary outcomes. We will now discuss how gaze to faces could influence vocabulary outcomes, pointing out how different strands of future research can tackle the further assessment of these cascading effects. We will then discuss some of the limitations of the review and point to possible future directions.

A number of studies related gaze to the eyes versus mouth to vocabulary outcomes. Studies examined both how infant gaze to the eyes (Carpenter et al., 1998; Mundy and Gomes, 1998; Morales et al., 2000; Brooks and Meltzoff, 2005, 2008) and to the mouth relates to vocabulary outcomes (Young et al., 2009; Elsabbagh et al., 2014; Tenenbaum et al., 2015), and how these relations change over development. That is, infants increasingly attend to the mouth over the course of the first year of life, and this developmental shift longitudinally predicts infants’ vocabulary outcomes later in development (Elsabbagh et al., 2014; Tenenbaum et al., 2015). Developing a capacity to switch between fixating to the eyes or mouth of a social partner is shown to have feedforward effects on vocabulary outcomes because the eyes and mouth provide meaningful yet different cues to word-object pairings in their environment. Arguably, infants who can selectively attend to the eyes and the mouth at points in time when such a facial feature is maximally socially informative will receive more cues to guide their word learning. Subsequently, these infants will have larger vocabulary outcomes compared to other infants. Yet, although there is substantial research linking gaze to the eyes or to mouth with word learning, additional clarification is needed on when and in which situations it is that infants develop an appropriate ability to socially encode information from the more relevant facial features, such that their gazes predicts and facilitates their word learning.

Whether there is a relationship between infants’ preference to gaze to faces (over other objects) and their vocabulary outcome remains theoretical, with no empirical evidence looking into whether face-preference has a feedforward effect on vocabulary outcomes. Additionally, no studies have attempted to correlate how infants’ preferential fixation to certain types of social faces may relate to their later vocabulary outcomes. Infant preference for faces (compared to objects) and their preference for more compared to less social faces may facilitate word learning by increasing the probability of infants attending to the relevant social cues that the face provides to guide their word learning (Tomasello, 1992, 2000).

Gaze to faces has frequently been theorized and empirically shown to have feedforward effects on vocabulary outcomes (Çetinçelik et al., 2021). Feedforward effects (also defined in the literature as cascading effects) are the cumulative consequences of the many interactions and transactions occurring in developing systems (Masten and Cicchetti, 2010; Sameroff, 2010; D’Souza and Karmiloff-Smith, 2011; Junge et al., 2020). Preferential gaze to faces is an early instance of selective attention, that may direct infants’ gazes to faces and provide infants with extensive experience of face stimuli. This extensive experience with faces could be a precursor to more complex social abilities (e.g., gaze following) that have frequently been shown to relate to vocabulary outcomes (Çetinçelik et al., 2021). Subsequently, infants’ early face preference could feed into word learning by feeding into infants’ complex social abilities that directly relate to their word learning. Understanding these feedforward effects of preferential gaze to faces on vocabulary outcomes may guide us toward the mechanisms and constraints leading to the acquisition of words and the subsequent vocabulary outcomes (D’Souza and Filippi, 2017; Kidd and Donnelly, 2020). In light of this, studies correlating infants’ early preferential gaze to faces with their vocabulary outcomes will give us insight into whether and why infants can learn their first words from the information that faces provide.

As yet, confirmative research is required to substantiate our hypotheses and further explain the nature of this feedforward effect. Regarding this nature, it is possible that gaze to faces has some direct feedforward effects on vocabulary outcomes. Alternatively, it is possible that face-preference serves as a mediator to vocabulary outcomes, as it scaffolds more sophisticated social abilities, such as joint attention (Junge et al., 2020). The strength of the relationship between infant gaze to faces and vocabulary outcomes could change as a function of the developmental stage, with face-preference facilitating word learning earlier in development more than later in development. The relation between gazes to faces and vocabulary outcomes is therefore likely to be stronger when assessing younger infants, because older infants have access to a larger range of social mechanisms, e.g., joint attention, that are made up of multiple smaller social domains. Therefore, we recommend that future research explores the relationship between the three domains that are addressed in this review (gazes to eyes and mouth; gazes to faces versus objects; and gazes to specific face types) and vocabulary outcomes in younger infant groups, who have fewer social capabilities at their disposal for word learning.

Additionally, the task given to the infant is also likely to affect the feedforward effect. When examining how infants attend to faces it is therefore important to consider both how the developmental stage and task given to the infant influence their processing of a face that they could attend to and how this processing may subsequently impact infants’ vocabulary outcomes.

### Limitations

This review has some limitations. First, as outlined above, the links made between some aspects of infant gaze to faces and vocabulary outcomes in this review remain theoretical and require more empirical evidence. Although we did not systematically review experimental findings, it appears there is insufficient research to draw clearer conclusions about how specific aspects of infant gaze to faces feed into vocabulary outcomes. Additional research is needed to confirm these links, including large-scale longitudinal studies, experimental studies with different paradigms, and intervention studies to illuminate whether, how, and when infant gaze to (aspects of) faces impacts vocabulary outcomes (Masten and Cicchetti, 2010).

Second, it is also important to take into account that the research from which we draw our theoretical links involve primarily (if not completely) samples of infants and parents from societies that are Western, educated, industrialized, rich, and democratic (that is, WEIRD societies: Henrich et al., 2010). The beliefs, traditions, and day-to-day lives of individuals from non-WEIRD societies may differ from WEIRD societies to the extent whereby the links observed in one society may not be comparable to the other. For example, whereas in Boston, MA, America (a WEIRD society) mothers are shown to frequently visually engage with their infants, in Gusii, Kenya (a non-WEIRD society) mothers engage more frequently through holding and touching instead of direct gazing at their infant (Akhtar and Gernsbacher, 2007). Our hypothesis that gaze to faces proves relevant in a number of ways should therefore not be interpreted as the most significant or only successful facilitators of word learning.

Our aim was to disentangle how an early developing social cue (infants’ attending to faces) related to vocabulary outcomes. Of course, this does not mean that gaze to faces is the only potential cue that relates to vocabulary outcomes. The literature documents a myriad of factors that impacts early word learning, ranging from the infant level to familial risks, to the environment (Kidd and Donnelly, 2020). For instance, at the infant level there exist many possible predictors: infants’ non-verbal cognitive skills (Colombo et al., 2008; Rose et al., 2009), general auditory abilities (Benasich and Tallal, 2002), and speech perception abilities (Fernald and Marchman, 2012; Cristia et al., 2014; Ference and Curtin, 2015; Wang et al., 2021). Moreover, we note that there are several other perceptual processes developing during infancy that contribute to more complex forms of social processing, such as joint attention (Lewkowicz and Ghazanfar, 2006; Scherf and Scott, 2012; Hadley et al., 2014; Happé and Frith, 2014; Pascalis et al., 2014). In this review, we have chosen to restrict the scope to fixation to faces as a proxy of early social behavior observable in infants from birth onward. It remains to be seen how all these potential factors hold together when explaining individual variations in early vocabulary.

### Future research directions

Based on the research findings and hypotheses compiled in this review, there are a number of gaps in the literature and subsequent directions that future research can take to further elucidate the role of infants attending to faces in being relevant to vocabulary outcomes. One line of studies could use repeated multiple measurements to investigate how and when across development gaze to faces (over objects) or gaze to more (compared to less) socially informative types of faces are indeed related to vocabulary outcomes. This could shed light on the developmental processes that gaze to faces comprises. For example, how infants attend to faces (compared to objects) may be more predictive of vocabulary outcomes earlier in development and become less predictive when infants start utilizing additional cues (such as direct and averted gaze) to guide their learning. Similarly, gaze to social faces may (or may not) become less predictive of vocabulary outcomes as infants start to direct more of their gaze to the surrounding environment or to other social stimuli, e.g., the hands (Smith and Yu, 2013; Deák et al., 2014; Jayaraman et al., 2015; Fausey et al., 2016).

Another line of research should focus on explaining the individual variation in early vocabulary (Bates et al., 1994; Frank et al., 2017). Understanding the sources of individual variation ultimately informs both theory-forming as well as practitioners aiming to maximize children’s word-learning potential (Kidd and Donnelly, 2020). To assess whether variation in early gaze to faces predicts word learning, as we hypothesize here, we need more empirical evidence testifying that there is such a link, and why such a link would exist. For example, it could well be that infants who look more at their partners may in turn receive more social responses from their partners that prolong the length of the interaction. Lengthening the interaction may increase the time window in which word learning can occur and improve the quality of the interaction may increase the probability of correct word-object pairings being made. Short-term, this could lead to more word learning opportunities when the infant is engaged in communication with a partner and long-term it could lead to observable differences in the vocabulary size and content of the infant. Subsequent studies could then use intervention-designs focusing on fostering infants’ fixation to faces to promote early vocabulary.

Finally, research could focus on well-controlled laboratory studies to carefully examine how the task within which the infant is engaged could impact the extent to which gaze to faces is predictive of vocabulary outcomes. For example, it has been shown that in some experimental settings, 1-year-olds hardly look at the faces of their social partner, but instead coordinate joint attention between themselves and a social partner by attending to objects held by themselves or their partner (Smith and Yu, 2013). In situations where the infant and the partner are handling the objects instead of looking to a faraway/out-of-reach object, there may be a lower ambiguity of the word-referent pairing, and thus less reliance on facial cues, than when an object being referred to is not in direct reach of the infant or social partner (Deák et al., 2014). Taking into account the interaction contexts (or tasks) in which infants learn words will expand our understanding of how and why visual social cues, such as those present in the faces of their social partners, affect word learning.

Future research could thus take into account how developmental, task-related and individual differences in infants attending to faces have feedforward effects on vocabulary outcomes. There are ample opportunities and directions for future research.

## Conclusion

Overall, infant gaze to faces could have an important effect on early word learning through the constrictions that facial cues provide on the natural variability of environments. A face-preference appears to be an initial bias that aids infants’ gazes to social stimuli early in development, when they have less attentional control. It may also feed into later developing, more complex social abilities, such as gaze following, that have been found to relate to word learning and vocabulary outcomes. Gazes to specific features of the face, on the other hand, develop over time and may constrict information relating to words, referents, and word-object pairings. Infants’ gazes to the eyes may aid in their discerning of communicative intent and in determining where in the environment the gaze of a social partner lies. Gaze to mouth movements provide multi-modal information to aid the processing of speech and learning of words, as well as reinforcing child-directed speech. Combined, these processes provide numerous cues to facilitate the creation of word-object pairings. There are a number of studies that have shown how infants gazes to the eyes and mouth relate to vocabulary outcomes. However, whether infant gaze to faces (compared to objects) as well as to more socially relevant types of faces relate to vocabulary outcomes remains speculative and could depend on an infant’s developmental level, which affects their ability to correctly discern and use such cues to guide their learning. Developmental level, as well as the task (i.e., situational factors), is therefore important to consider when evaluating correlations between infant gaze to faces and their vocabulary outcomes. Although this review hypothesizes that infant gaze to faces relates to their vocabulary outcomes, and finds some evidence in favor of our hypothesis, future empirical studies could examine the feedforward effects on vocabulary outcomes more directly.

## Author contributions

ZB, CB, and CJ: conceptualization, investigation, writing—original draft, and writing—review and editing. CB and CJ: funding acquisition and supervision. ZB: project administration. All authors contributed to the article and approved the submitted version.
